# 5-(4-Ethoxy­benzyl)-1*H*-tetra­zole

**DOI:** 10.1107/S1600536809055597

**Published:** 2010-01-09

**Authors:** Yun-Long Gao, Gui-Long Zhao, Hua Shao, Wei Liu, Jian-wu Wang

**Affiliations:** a School of Chemistry and Chemical Engineering, Shandong University, Jinan 250100, People’s Republic of China; b Tianjin Key Laboratory of Molecular Design and Drug Discovery, Tianjin Institute of Pharmaceutical Research, Tianjin 300193, People’s Republic of China

## Abstract

In the title mol­ecule, C_10_H_12_N_4_O, the tetra­zole and benzene rings form a dihedral angle of 67.52 (2)°. In the crystal, inter­molecular N—H⋯N hydrogen bonds link the mol­ecules into chains along the *a* axis. The relatively short distance of 3.760 (3) Å between the centroids of the tetra­zole rings suggests the existence of π–π inter­actions.

## Related literature

For details of the biological activities of sodium-glucose co-transporter 2 (SGLT2) inhibitors, see: Arakawa *et al.* (2001 ▶); Meng *et al.* (2008 ▶). For bond-length data, see: Allen *et al.* (1987 ▶).
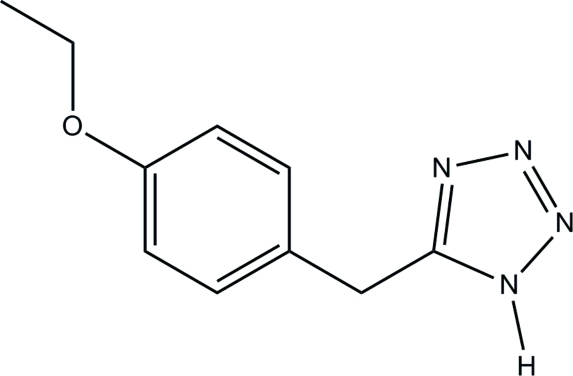


## Experimental

### 

#### Crystal data

C_10_H_12_N_4_O
*M* *_r_* = 204.24
Monoclinic, 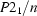

*a* = 4.9291 (10) Å
*b* = 18.145 (4) Å
*c* = 11.363 (2) Å
β = 99.19 (3)°
*V* = 1003.2 (3) Å^3^
*Z* = 4
Mo *K*α radiation
μ = 0.09 mm^−1^
*T* = 113 K
0.34 × 0.06 × 0.04 mm

#### Data collection

Rigaku Saturn CCD area-detector diffractometer
Absorption correction: multi-scan (*CrystalClear*; Rigaku, 2007 ▶) *T*_min_ = 0.969, *T*_max_ = 0.996
6870 measured reflections
1768 independent reflections
1487 reflections with *I* > 2σ(*I*)
*R*_int_ = 0.048

#### Refinement

*R*[*F* ^2^ > 2σ(*F* ^2^)] = 0.043
*wR*(*F* ^2^) = 0.103
*S* = 1.10
1768 reflections
142 parameters
1 restraint
H atoms treated by a mixture of independent and constrained refinement
Δρ_max_ = 0.24 e Å^−3^
Δρ_min_ = −0.34 e Å^−3^

### 

Data collection: *CrystalClear* (Rigaku, 2007 ▶); cell refinement: *CrystalClear*; data reduction: *CrystalClear*; program(s) used to solve structure: *SHELXS97* (Sheldrick, 2008 ▶); program(s) used to refine structure: *SHELXL97* (Sheldrick, 2008 ▶); molecular graphics: *SHELXTL* (Sheldrick, 2008 ▶); software used to prepare material for publication: *SHELXTL*.

## Supplementary Material

Crystal structure: contains datablocks I, global. DOI: 10.1107/S1600536809055597/cv2684sup1.cif

Structure factors: contains datablocks I. DOI: 10.1107/S1600536809055597/cv2684Isup2.hkl

Additional supplementary materials:  crystallographic information; 3D view; checkCIF report

## Figures and Tables

**Table 1:** Hydrogen-bond geometry (Å, °)

*D*—H⋯*A*	*D*—H	H⋯*A*	*D*⋯*A*	*D*—H⋯*A*
N1—H1⋯N4^i^	0.91 (1)	1.90 (1)	2.7897 (16)	166 (1)

Symmetry code: (i) 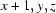.

**Fig. 1.**
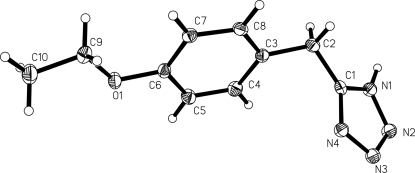
The molecular structure of (I) with the atomic labels and 40% probability displacement ellipsoids.

## supplementary crystallographic information

### Comment

Sodium-Glucose Cotransporter 2 (SGLT2) inhibitors constitute a new class of antidiabetic agents (Arakawa *et al.*, 2001; Meng *et al.*, 2008). The title compound, (I), was prepared as an intermediate of a new class of SGLT2 inhibitors designed in our laboratories.

In (I) (Fig. 1), all bond lengths in the molecular are normal (Allen *et al.*, 1987). Atoms O1/C2/C9/C10 lie in the benzene ring (C3—C8) plane with a maximun deviation of 0.045 (2) Å for O1 . The tetrazole ring (N1—N4/C1) forms the dihedral angle of 67.52 (2) ° with the benzene ring (C3—C8).

In the crystal structure, relatively short distance of 3.760 (3) Å between the centroids of tetrazole rings suggests an existence of π—π interactions. Intermolecular N—H···N hydrogen bonds (Table 1) link the molecules related by translation along axis *a* into chains.

### Experimental

A round-bottomed flask was charged with 1.61 g (10 mmol) of 4-ethoxyphenylacetonitrile, 3.25 g (50 mmol) of sodium azide and 2.67 g (50 mmol) of ammonium chloride and 50 ml of DMF, and the resulting mixture was stirred at 120 ° C for 15 h. On complete cooling, the mixture was filtered to remove the existing solid and the filtrate was evaporated on a rotary evaporator equipped with an oil pump, and the residue was dissolved in 100 ml of water. The aqueous solution thus obtained was adjusted to pH = 2 with concentrated hydrochloric acid, when it turned turbid. This turbid mixture was cooled with ice-water bath and stirred for complete crystallization. The precipitated crystals were collected *via* suction filtration and dried at 60° C *in vacuo* to afford the title product as white crystals 1.68 g (yield 82.3%). Crystals suitable for single-crystal X-ray diffraction were obtained *via* slow evaporation at room temperature of a solution of the pure title compound in dichloromethane/petroleum ether.

### Refinement

All H atoms were found on difference maps. C-bound H atoms were placed in idealized positions (C—H 0.93 - 0.97 Å),  and included in the final cycles of refinement using a riding model, with *U*_iso_(H) = 1.2*U*_eq_(C) for aryl and methylene and 1.5*U*_eq_(C) for the methyl H atoms. The N-bound H atom was refined isotropically.

### Crystal data

**Table:**

C_10_H_12_N_4_O	*F*(000) = 432
*M**_r_* = 204.24	*D*_x_ = 1.352 Mg m^−^^3^
Monoclinic, *P*2_1_/*n*	Mo *K*α radiation, λ = 0.71073 Å
Hall symbol: -P 2yn	Cell parameters from 3598 reflections
*a* = 4.9291 (10) Å	θ = 1.1–27.9°
*b* = 18.145 (4) Å	µ = 0.09 mm^−^^1^
*c* = 11.363 (2) Å	*T* = 113 K
β = 99.19 (3)°	Needle, colourless
*V* = 1003.2 (3) Å^3^	0.34 × 0.06 × 0.04 mm
*Z* = 4	

### Data collection

**Table:**

Rigaku Saturn CCD area-detector diffractometer	1768 independent reflections
Radiation source: rotating anode	1487 reflections with *I* > 2σ(*I*)
multilayer	*R*_int_ = 0.048
Detector resolution: 14.63 pixels mm^-1^	θ_max_ = 25.0°, θ_min_ = 2.1°
ω and φ scans	*h* = −5→5
Absorption correction: multi-scan (*CrystalClear*; Rigaku, 2007)	*k* = −21→19
*T*_min_ = 0.969, *T*_max_ = 0.996	*l* = −13→13
6870 measured reflections	

### Refinement

**Table:**

Refinement on *F*^2^	Secondary atom site location: difference Fourier map
Least-squares matrix: full	Hydrogen site location: inferred from neighbouring sites
*R*[*F*^2^ > 2σ(*F*^2^)] = 0.043	H atoms treated by a mixture of independent and constrained refinement
*wR*(*F*^2^) = 0.103	*w* = 1/[σ^2^(*F*_o_^2^) + (0.067*P*)^2^] where *P* = (*F*_o_^2^ + 2*F*_c_^2^)/3
*S* = 1.10	(Δ/σ)_max_ = 0.004
1768 reflections	Δρ_max_ = 0.24 e Å^−^^3^
142 parameters	Δρ_min_ = −0.34 e Å^−^^3^
1 restraint	Extinction correction: *SHELXL97* (Sheldrick, 2008), Fc^*^=kFc[1+0.001xFc^2^λ^3^/sin(2θ)]^-1/4^
Primary atom site location: structure-invariant direct methods	Extinction coefficient: 0.354 (18)

### Special details

**Table:**

Geometry. All e.s.d.'s (except the e.s.d. in the dihedral angle between two l.s. planes) are estimated using the full covariance matrix. The cell e.s.d.'s are taken into account individually in the estimation of e.s.d.'s in distances, angles and torsion angles; correlations between e.s.d.'s in cell parameters are only used when they are defined by crystal symmetry. An approximate (isotropic) treatment of cell e.s.d.'s is used for estimating e.s.d.'s involving l.s. planes.
Refinement. Refinement of *F*^2^ against ALL reflections. The weighted *R*-factor *wR* and goodness of fit *S* are based on *F*^2^, conventional *R*-factors *R* are based on *F*, with *F* set to zero for negative *F*^2^. The threshold expression of *F*^2^ > σ(*F*^2^) is used only for calculating *R*-factors(gt) *etc*. and is not relevant to the choice of reflections for refinement. *R*-factors based on *F*^2^ are statistically about twice as large as those based on *F*, and *R*- factors based on ALL data will be even larger.

### Fractional atomic coordinates and isotropic or equivalent isotropic displacement parameters (Å^2^)

**Table:**

	*x*	*y*	*z*	*U*_iso_*/*U*_eq_	
O1	0.09591 (18)	0.17311 (5)	0.48558 (8)	0.0241 (3)	
N1	1.0194 (2)	0.48750 (6)	0.33955 (10)	0.0214 (3)	
N2	0.9379 (2)	0.55573 (6)	0.36698 (11)	0.0252 (3)	
N3	0.6749 (2)	0.55229 (5)	0.36251 (11)	0.0242 (3)	
N4	0.5824 (2)	0.48270 (5)	0.33271 (10)	0.0211 (3)	
C1	0.8013 (2)	0.44332 (7)	0.31894 (11)	0.0187 (3)	
C2	0.8099 (3)	0.36438 (7)	0.28353 (12)	0.0225 (4)	
H2A	0.9937	0.3456	0.3100	0.027*	
H2B	0.7744	0.3613	0.1972	0.027*	
C3	0.6058 (3)	0.31536 (7)	0.33333 (12)	0.0195 (3)	
C4	0.5715 (3)	0.32048 (7)	0.45271 (12)	0.0234 (3)	
H4	0.6676	0.3562	0.5012	0.028*	
C5	0.3974 (3)	0.27333 (7)	0.49957 (12)	0.0243 (4)	
H5	0.3739	0.2781	0.5788	0.029*	
C6	0.2566 (2)	0.21864 (7)	0.42871 (12)	0.0198 (3)	
C7	0.2858 (2)	0.21280 (7)	0.30957 (11)	0.0204 (3)	
H7	0.1916	0.1766	0.2614	0.024*	
C8	0.4583 (3)	0.26196 (7)	0.26324 (12)	0.0206 (3)	
H8	0.4748	0.2588	0.1830	0.025*	
C9	−0.0624 (3)	0.11821 (7)	0.41507 (12)	0.0244 (4)	
H9A	0.0578	0.0838	0.3832	0.029*	
H9B	−0.1819	0.1410	0.3490	0.029*	
C10	−0.2311 (3)	0.07851 (8)	0.49534 (14)	0.0319 (4)	
H10A	−0.1106	0.0560	0.5601	0.048*	
H10B	−0.3407	0.0412	0.4505	0.048*	
H10C	−0.3489	0.1131	0.5264	0.048*	
H1	1.197 (2)	0.4782 (8)	0.3340 (13)	0.030 (4)*	

### Atomic displacement parameters (Å^2^)

**Table:**

	*U*^11^	*U*^22^	*U*^33^	*U*^12^	*U*^13^	*U*^23^
O1	0.0280 (6)	0.0230 (5)	0.0223 (6)	−0.0068 (4)	0.0069 (4)	0.0006 (4)
N1	0.0145 (6)	0.0227 (6)	0.0272 (7)	0.0000 (5)	0.0042 (5)	0.0004 (5)
N2	0.0213 (6)	0.0226 (6)	0.0322 (7)	−0.0013 (5)	0.0056 (5)	−0.0012 (5)
N3	0.0212 (6)	0.0227 (6)	0.0292 (7)	−0.0017 (5)	0.0062 (5)	−0.0001 (5)
N4	0.0174 (6)	0.0204 (6)	0.0255 (7)	−0.0009 (5)	0.0037 (5)	−0.0005 (4)
C1	0.0161 (7)	0.0232 (7)	0.0170 (7)	−0.0019 (5)	0.0033 (5)	0.0031 (5)
C2	0.0184 (7)	0.0232 (7)	0.0270 (8)	0.0011 (5)	0.0066 (6)	−0.0001 (5)
C3	0.0165 (7)	0.0185 (7)	0.0239 (8)	0.0030 (5)	0.0042 (6)	0.0025 (5)
C4	0.0241 (8)	0.0220 (7)	0.0232 (8)	−0.0034 (5)	0.0009 (6)	−0.0033 (5)
C5	0.0292 (8)	0.0262 (7)	0.0177 (8)	−0.0028 (6)	0.0047 (6)	−0.0009 (5)
C6	0.0184 (7)	0.0182 (7)	0.0228 (8)	0.0006 (5)	0.0034 (6)	0.0033 (5)
C7	0.0200 (7)	0.0195 (7)	0.0211 (8)	−0.0007 (5)	0.0017 (6)	−0.0020 (5)
C8	0.0222 (7)	0.0220 (7)	0.0183 (8)	0.0036 (5)	0.0055 (6)	−0.0001 (5)
C9	0.0237 (7)	0.0204 (7)	0.0284 (9)	−0.0029 (6)	0.0022 (6)	0.0011 (5)
C10	0.0268 (8)	0.0277 (8)	0.0418 (10)	−0.0040 (6)	0.0077 (7)	0.0055 (6)

### Geometric parameters (Å, °)

**Table:**

O1—C6	1.3753 (15)	C4—C5	1.3778 (18)
O1—C9	1.4296 (16)	C4—H4	0.9300
N1—C1	1.3313 (16)	C5—C6	1.3914 (18)
N1—N2	1.3533 (15)	C5—H5	0.9300
N1—H1	0.906 (9)	C6—C7	1.3881 (18)
N2—N3	1.2908 (16)	C7—C8	1.3922 (18)
N3—N4	1.3666 (14)	C7—H7	0.9300
N4—C1	1.3244 (16)	C8—H8	0.9300
C1—C2	1.4903 (18)	C9—C10	1.5113 (18)
C2—C3	1.5184 (17)	C9—H9A	0.9700
C2—H2A	0.9700	C9—H9B	0.9700
C2—H2B	0.9700	C10—H10A	0.9600
C3—C8	1.3845 (18)	C10—H10B	0.9600
C3—C4	1.3965 (18)	C10—H10C	0.9600
			
C6—O1—C9	117.34 (10)	C4—C5—H5	119.9
C1—N1—N2	109.29 (10)	C6—C5—H5	119.9
C1—N1—H1	129.7 (10)	O1—C6—C7	124.72 (12)
N2—N1—H1	120.9 (10)	O1—C6—C5	115.34 (11)
N3—N2—N1	106.26 (10)	C7—C6—C5	119.94 (12)
N2—N3—N4	110.35 (10)	C6—C7—C8	118.98 (12)
C1—N4—N3	106.37 (10)	C6—C7—H7	120.5
N4—C1—N1	107.74 (11)	C8—C7—H7	120.5
N4—C1—C2	127.53 (11)	C3—C8—C7	121.81 (12)
N1—C1—C2	124.71 (11)	C3—C8—H8	119.1
C1—C2—C3	114.42 (10)	C7—C8—H8	119.1
C1—C2—H2A	108.7	O1—C9—C10	107.31 (11)
C3—C2—H2A	108.7	O1—C9—H9A	110.3
C1—C2—H2B	108.7	C10—C9—H9A	110.3
C3—C2—H2B	108.7	O1—C9—H9B	110.3
H2A—C2—H2B	107.6	C10—C9—H9B	110.3
C8—C3—C4	118.14 (11)	H9A—C9—H9B	108.5
C8—C3—C2	120.93 (12)	C9—C10—H10A	109.5
C4—C3—C2	120.86 (12)	C9—C10—H10B	109.5
C5—C4—C3	120.89 (12)	H10A—C10—H10B	109.5
C5—C4—H4	119.6	C9—C10—H10C	109.5
C3—C4—H4	119.6	H10A—C10—H10C	109.5
C4—C5—C6	120.20 (13)	H10B—C10—H10C	109.5
			
C1—N1—N2—N3	−0.13 (14)	C2—C3—C4—C5	−176.44 (11)
N1—N2—N3—N4	0.10 (14)	C3—C4—C5—C6	1.3 (2)
N2—N3—N4—C1	−0.03 (14)	C9—O1—C6—C7	−3.22 (17)
N3—N4—C1—N1	−0.05 (14)	C9—O1—C6—C5	177.21 (11)
N3—N4—C1—C2	−178.47 (12)	C4—C5—C6—O1	177.84 (11)
N2—N1—C1—N4	0.12 (15)	C4—C5—C6—C7	−1.75 (19)
N2—N1—C1—C2	178.59 (12)	O1—C6—C7—C8	−179.16 (11)
N4—C1—C2—C3	−36.43 (19)	C5—C6—C7—C8	0.39 (18)
N1—C1—C2—C3	145.41 (13)	C4—C3—C8—C7	−1.88 (18)
C1—C2—C3—C8	138.01 (13)	C2—C3—C8—C7	175.05 (11)
C1—C2—C3—C4	−45.14 (17)	C6—C7—C8—C3	1.45 (19)
C8—C3—C4—C5	0.49 (19)	C6—O1—C9—C10	−177.01 (10)

### Hydrogen-bond geometry (Å, °)

**Table:**

*D*—H···*A*	*D*—H	H···*A*	*D*···*A*	*D*—H···*A*
N1—H1···N4^i^	0.91 (1)	1.90 (1)	2.7897 (16)	166 (1)

Symmetry codes: (i) *x*+1, *y*, *z*.
